# Spike-in normalization for single-cell RNA-seq reveals dynamic global transcriptional activity mediating anticancer drug response

**DOI:** 10.1093/nargab/lqab054

**Published:** 2021-06-17

**Authors:** Xin Wang, Jane Frederick, Hongbin Wang, Sheng Hui, Vadim Backman, Zhe Ji

**Affiliations:** Department of Pharmacology, Feinberg School of Medicine, Northwestern University, Chicago, IL 60611, USA; Department of Biomedical Engineering, McCormick School of Engineering, Northwestern University, Evanston, IL 60628, USA; Department of Pharmacology, Feinberg School of Medicine, Northwestern University, Chicago, IL 60611, USA; Department of Molecular Metabolism, Harvard T. H. Chan School of Public Health, 655 Huntington Avenue, Boston, MA 02115, USA; Department of Biomedical Engineering, McCormick School of Engineering, Northwestern University, Evanston, IL 60628, USA; Department of Pharmacology, Feinberg School of Medicine, Northwestern University, Chicago, IL 60611, USA; Department of Biomedical Engineering, McCormick School of Engineering, Northwestern University, Evanston, IL 60628, USA

## Abstract

The transcriptional plasticity of cancer cells promotes intercellular heterogeneity in response to anticancer drugs and facilitates the generation of subpopulation surviving cells. Characterizing single-cell transcriptional heterogeneity after drug treatments can provide mechanistic insights into drug efficacy. Here, we used single-cell RNA-seq to examine transcriptomic profiles of cancer cells treated with paclitaxel, celecoxib and the combination of the two drugs. By normalizing the expression of endogenous genes to spike-in molecules, we found that cellular mRNA abundance shows dynamic regulation after drug treatment. Using a random forest model, we identified gene signatures classifying single cells into three states: transcriptional repression, amplification and control-like. Treatment with paclitaxel or celecoxib alone generally repressed gene transcription across single cells. Interestingly, the drug combination resulted in transcriptional amplification and hyperactivation of mitochondrial oxidative phosphorylation pathway linking to enhanced cell killing efficiency. Finally, we identified a regulatory module enriched with metabolism and inflammation-related genes activated in a subpopulation of paclitaxel-treated cells, the expression of which predicted paclitaxel efficacy across cancer cell lines and *in vivo* patient samples. Our study highlights the dynamic global transcriptional activity driving single-cell heterogeneity during drug response and emphasizes the importance of adding spike-in molecules to study gene expression regulation using single-cell RNA-seq.

## INTRODUCTION

A major challenge of cancer therapy is the acquired resistance of cancer cells to chemotherapy drugs and associated disease relapse. A driver of drug resistance is intercellular heterogeneity of gene expression resulting from genetic mutations and epigenetic alterations (1,2). The pre-existing and rewired gene expression programs of cancer cells determine their ultimate fates after drug treatment. Emerging evidence shows that even genetically identical cells show transcriptional heterogeneity in response to external stimuli under both physiological and *in vitro* conditions because of their transcriptional plasticity (3,4). Characterizing single-cell transcriptomic dynamics after drug treatment can provide mechanistic insights into cell fate decisions and identify predictive biomarkers of efficacy (5).

Paclitaxel is a commonly used chemotherapy drug for treating diverse human cancers, such as ovarian, breast and lung cancers. It binds to the microtubule polymer and disrupts its disassembly, which triggers mitotic arrest and apoptosis (6). However, most patients develop chemoresistance after several sessions of treatment. Many studies have been devoted to identifying the molecular mechanisms mediating paclitaxel resistance and developing combinatory therapeutic strategies (7,8). For example, synergistic inhibition of NF-κB and PI3K signaling pathways can sensitize paclitaxel-resistant cancer cells (9,10). Combination treatment with the nonsteroidal anti-inflammatory drug celecoxib (a COX-2 inhibitor) can increase paclitaxel’s efficacy at killing cancer cells (11–13).

Despite extensive effort, the molecular mechanisms regulating paclitaxel efficacy remain elusive. A limitation is that many studies were carried out using bulk cancer cells, without considering the contribution of intercellular heterogeneity. Recently, using partial wave spectroscopic (PWS) microscopy, we observed that treatment with paclitaxel increases the packing density scaling of chromatin domains and their intercellular heterogeneity in surviving subpopulations of cancer cells, suggesting that surviving cells exhibit a phenotype consistent with enhanced single-cell transcriptional heterogeneity (14). On the other hand, celecoxib treatment decreases the chromatin packing density scaling within chromatin domains of cells. Due to the opposite effects of paclitaxel and celecoxib on chromatin packing in the nucleus observed from our imaging experiments (14), we hypothesized that their combination may induce a *de novo* transcriptional program promoting cancer cell death.

To obtain molecular insights into the drug response, we performed full-length single-cell RNA sequencing (scRNA-seq) with Smart-seq2 (15) and profiled the transcriptomes of several hundred single cells at different time points after treatment with paclitaxel, celecoxib and the combination of the two drugs, respectively. To compare the global transcriptomic levels across single cells, we added a constant amount of polyA-tailed spike-in molecules to each cell during the library preparation (16). By normalizing endogenous gene expression levels to those of spike-in molecules, we found that global transcriptomic levels are heterogeneous across single cells and are dynamically regulated after drug treatment.

Paclitaxel treatment alone generally repressed transcriptomic levels, while its combination with celecoxib resulted in transcriptional amplification. We developed a random forest model and classified single cells based on their transcriptional states. The model revealed that the downregulation of the cell cycle pathway is associated with transcriptional repression, and the hyperactivation of mitochondrial oxidative phosphorylation (OXPHOS) contributes to the transcriptional amplification and enhanced cell killing efficacy after the drug combination. Furthermore, we identified a coherent gene module regulating cellular metabolism and inflammation, the higher expression of which predicts worse paclitaxel response in cancer cell lines and patients. Altogether, our study highlights the unprecedented effects of single-cell heterogeneity on global transcriptional activity in regulating the anticancer drug response.

## MATERIALS AND METHODS

### Cell culture

A2780 ovarian endometroid adenocarcinoma cells were a gift from Dr Chia-Peng Huang Yang at the Albert Einstein College of Medicine obtained from Dr Elizabeth de Vries at the University Medical Center Groningen. The cells were cultured in RPMI 1640 medium (Thermo Fisher Scientific, Waltham, MA) supplemented with 10% fetal bovine serum (Thermo Fisher Scientific, Waltham, MA) on 35-mm six-well glass bottom plates (Cellvis, Mountain View, CA) until 60–85% confluent. All cells were given at least 24 h to read here before drug treatment.

### Cell growth and apoptosis experiments

Cells were treated with 75 μM celecoxib, 5 nM paclitaxel, or a combination of 75 μM celecoxib and 5 nM paclitaxel for 48 h. Cells were then imaged to determine the percent coverage of the well for each treatment. To determine the amount of cell growth inhibition based on a treatment, the amount of cell coverage was normalized to the control group. Apoptosis staining was performed using the CellEvent Caspase-3/7 Green Flow Cytometry Assay Kit (Thermo Fisher Scientific, Waltham, MA). Stained cell suspensions were measured with the BD LSRFortessa Cell Analyzer (BD Biosciences, San Jose, CA) at the Northwestern University Flow Cytometry Core Facility.

### scRNA-seq Library preparation using SMART-seq2

Cells were treated with 75 μM celecoxib for 2 and 16 h, 5 nM paclitaxel for 16 and 48 h, or a combination of 75 μM celecoxib and 5 nM paclitaxel for 16 h prior to trypsinization and resuspension in growth medium. Cell suspensions were sorted with a C1 single-cell capture system (Fluidigm, South San Francisco, CA) by the University of Illinois at Chicago Genomics Core. scRNA-seq libraries were prepared according to the Smart-seq2 protocol and sequenced using the NextSeq 500 Sequencing System (Illumina, San Diego, CA) by the University of Illinois at Chicago Sequencing Core. A predesigned set of three polyA-tailed spike-in RNAs (sequences are shown in Supplementary Table S1) was added to each well during the library preparation.

### scRNA-seq data processing

We trimmed the adapter sequences of raw sequencing reads using the Trim Galore software (https://www.bioinformatics.babraham.ac.uk/projects/trim_galore/). We used RSEM (17) to calculate RNA expression levels using scRNA-seq data. We created an RSEM index with the combination of the human hg38 transcriptome (GENCODE version 28 (18)) and 3 spike-in molecules. We calculated RNA expression using RSEM 1.3.0 with the default parameters and Bowtie2 (19) as the aligner. We normalized the expression levels of endogenous genes to that of the highest expressed spike-in molecule (16,20). Given *M* single cells, to control the dynamic regulation of endogenous gene expression in single cell *k*, the ‘transcript per million’ (TPM) value of gene *j* is normalized to that of spike-in 1 (*s*), which is the highest spike-in molecule. The normarlized expression of gene *j* in the cell *k* (${N_{jk}}$) was calculated as:


\begin{equation*}{N_{jk}} = TP{M_{jk}}\;/\left(TP{M_{sk}}/\left(\left( {\mathop \sum \limits_{i\; = \;1}^M TP{M_{si}}}\right)/M\right) \right)\end{equation*}

As the RNA capturing complexity of the scRNA-seq library is ∼10% of bulk RNA-seq (21), we divided the above-normalized TPM values by 10, and the expression level of gene *j* in cell *k* was quantified as the *E*-value *E_jk_*:


\begin{equation*}\;{E_{jk}} = \;lo{g_2}\left( {\frac{{{N_{jk}}}}{{10}} + 1} \right)\end{equation*}

We performed quality-control of the scRNA-seq data and filtered out cells with housekeeping genes poorly detected (averaged *E*-value of the genes <1) (22). The housekeeping genes were defined in (23). We only included genes detected in >30% of single cells in at least one experimental condition in further analyses.

### Examining intercellular expression variance within each treatment group

To identify genes showing high intercellular expression variance within each treatment group, we calculated the log10 (coefficient of variation (CV)) for each gene (24,25). CV was measured as the ratio of the standard deviation of *E*-values across single cells to the mean. We then separated genes into 100 bins based on the mean *E*-values and calculated the ‘standard deviation of log10(CV)’ for each bin. Genes showing >2-fold of the ‘standard deviation of log10(CV)’ than those in the same bin were considered to have high intercellular expression variance. We used only highly expressed genes with mean *E*-values > 1 for the analyses.

### Principal component analysis and random forest modeling to classify the cell states

We used the averaged *E*-values of a gene across single cells to indicate its expression upon drug treatment. We selected 6175 genes showing differential expression after drug treatment (>1.5-fold change compared to control cells) for the principal component analysis and building the random forest model. For each gene, the control-normalized *E*-values were used as the input for the analyses.

To examine whether single cells from a drug treatment group are homogenous with unique gene signatures, we used the random forest model and associated out-of-bag estimates to measure the prediction power to classify the single cells from each drug treatment group versus those from other groups. The out-of-bag analyses were done using the R package ‘randomForest’ with the following parameters: ntree = 600, importance = TRUE, do.trace = 100, proximity = TRUE, keep.inbag = TRUE, and 6 classes (each treatment condition represents one class). Based on the out-of-bag analysis results, we used 200 trees for the downstream classification analyses.

To further classify the single cells based on their transcriptomic states, we used the control cells, cells treated with celecoxib for 16 h, and cells treated with both paclitaxel and celecoxib to represent the control-like, transcriptional repression and activation states, respectively. We randomly selected 2/3 of single cells from these treatment conditions to train the model with the following parameters: ntree = 200, importance = TRUE, do.trace = 100, proximity = TRUE, keep.inbag = TRUE, and 5-fold cross-validation. The classification of the remaining 1/3 of the cells was used to measure the algorithm performance using the receiver operating characteristic (ROC) curves. Genes with a mean decrease in accuracy (MDA) value >0 from the model contributed significantly to the classification. Finally, the model was used to classify all single cells into all three states with three corresponding *P*-values. The sum of the three *P*-values was 1, and the state with the largest *P*-value was defined as the classified state.

To further test the robustness of our classification, we used a different method to select single cells for the training set based on the PCA results. We selected control-like cells as ‘PC2 > 15’, cells with transcriptional repression as ‘PC1 > 15 & PC2 < 0 & PC3 > 5’, and these with transcription amplification as ‘PC1 < -15 & PC2 < 0’. This selection method did not restrict the preexisting cell labels. We randomly selected 25 single cells from each group, trained the random forest model, and applied the model to classify single cell states. We then calculated the fraction of single cells showing consistent classified states from this new method versus these from the model described in the last paragraph. We repeated the process 200 times to get the median value and 90% confidence interval.

### Differential gene expression and pathway analysis

We grouped regulated genes after drug treatments into three coherent clusters based on their relative differential expression changes after drug treatment. We used the average *E*-values across single cells in a drug treatment condition to indicate the expression level. About 1389 genes in cluster I were down-regulated >1.5-fold at 16 h of celecoxib treatment and up-regulated >1.5-fold at 16 h of combination drug treatment. About 2852 genes in cluster II were down-regulated at 16 h of celecoxib treatment and showed no dynamic expression change after combination treatment. About 1547 genes in cluster III were up-regulated >1.5-fold after combination treatment and showed no dynamic regulation after celecoxib treatment. These three clusters effectively captured 94% of all dynamically regulated genes in five different drug treatment conditions. The gene ontology analyses were performed using the Database for Annotation, Visualization and Integrated Discovery (DAVID) (26).

For a geneset regulating a biological process, we compared its expression levels across single cells after each drug treatment. Taking the OXPHOS pathway for example, we used the expressed genes in GO:0006119 for the analyses. Suppose there are *G* genes in the geneset. For each gene *j*, we first normalized its *E*-value in cell *k* to the mean of the control cells as *M_jk_*. The relative expression of the geneset in cell *k* was then calculated as the mean(*M_jk_*) *_j = 1, 2__…__G_*.

Using this method, we calculated the relative expression of genesets encoding complex I (GO:0005747), complex II (GO:0005749), complex III (GO:0005750), complex IV (GO:0005751) and ATP synthase of the OXPHOS pathway. We used genes in GO:0007049 for the analyses of cell cycle regulation. For genes showing unique activation in a particular cell cycle phase, we used previously defined gene lists (27). To compare the relative expression of a set of metabolism genes, we used the following paclitaxel-activated genes in the GO:0055114: oxidation-reduction process geneset for analysis, including TM7SF2, PHYHD1, ACADSB, HMGCR, HSD17B14, ALDH1A1, MTHFD2, TP53I3, PLOD1, NOS3, CCS, GPX8, HSD17B8, PTGR1, PTGR2, DHRS12, SCD, FADS1, DECR2, PHYH, VAT1, MSRB2, COQ6, BLVRA, SQLE, ALDH2, PHGDH, ACAD11.

### Calculation of the paclitaxel response index

We downloaded the RNA expression data measured by Affymetrix microarray and the corresponding annotations of 1037 cancer cell lines from the Cancer Cell Line Encyclopedia (CCLE) (28). Based on the averaged *E*-values across single cells, 177 genes were up-regulated (>1.5-fold) in the control-like cell subpopulation after 48 h of paclitaxel treatment. As these genes are co-activated after paclitaxel treatment, we next examined whether they form a coherent transcriptional module and their baseline expression levels are correlated across cancer cell lines. Using the CCLE data and an iterative computational approach, we identified 73 genes with a significant positive correlation of expression. The approach is as follows.

We analyzed the RNA expression levels of the 177 paclitaxel-activated genes using CCLE data. First, we required that a gene included for further analysis should be expressed in >50% of cancer cells and show variable expression levels across all 1037 cancer cells. We used the MAS5 algorithm to determine whether a gene is expressed in a cell. The difference between the 90th percentile and the 10th percentile of the gene was required to be >3-fold. This step filtered out genes showing lineage-specific or constitutive expression across cancer cells.

Second, for the geneset consisting of remaining genes from the above step, we calculated its relative expression across all 1037 cancer cells using a similar method as described above for the pathway analyses. Given *G* genes in the geneset, for each gene *j*, we first normalized its log2-based expression level in cell *k* to the median of all cancer cells as *M_jk_*, and the relative expression of the geneset in cell *k* was calculated as *S_k_* = median(*M_jk_*)*_j = 1, 2__…__G_*.

Third, we calculated the Spearman correlation coefficients between the geneset expression *S_k_* and the expression levels of each gene *j* across all 1037 cancer cells. We removed genes with a coefficient value <0.1 from the geneset. Using an iterative repetition of steps 2 and 3, we obtained 73 genes showing a significant positive correlation with each other. We then calculated the relative expression of the entire set of 73 genes as the paclitaxel response index across cancer cell lines. The 73 genes include SLC25A21, PTGR2, SCD, ALDH1A1, PLTP, ALDH2, FKBP1B, TP53I3, SERPING1, C6orf1, VAT1, PLOD1, IFI27L2, ARMCX3, BLVRA, SPA17, EFHC1, SBF2, RAB13, CDC42EP5, TNNC1, FBXO2, TLCD1, PLEKHA1, SAT1, SEMA3E, USP32, ABI2, PHYHD1, MYL5, DHRS12, OCEL1, HMGCL, CORO1B, GGPS1, ITM2B, NOS3, DECR2, ECI1, C9orf16, NEK3, SUCLG2, CRYL1, TST, ACER3, SERPINB6, CD46, YIPF3, SUMF2, SIL1, GRN, MSRB2, PHYH, TNFRSF10B, ETV4, DUSP6, S100A4, IFITM1, IFITM2, IFITM3, CA2, GJA1, IFI35, RAB32, LGALS1, HSPA1A, MYO1B, GPX8, ANXA1, PTGR1, RRAS, TNFRSF1A, S100A11.

### Cancer patient data analysis

We downloaded two clinical cohorts of breast cancer patient data from the NCBI Gene Expression Omnibus (GEO) database, including GSE25066 (Hatzis dataset (29)) and GSE32646 (Miyake dataset (30)). We used the R package affxparser to read and analyze RNA expression levels measured by the microarray data. Because the analysis is for breast cancer patients and RNA expression represents the averaged signals across cancer cells and stromal cells, we required that genes used to calculate the paclitaxel response index should be expressed in >50% of CCLE breast cancer cell lines. We used the following 51 genes to calculate the paclitaxel response index in breast cancer patients: S100A11, GRN, HSPA1A, PLOD1, ANXA1, LGALS1, IFITM2, ALDH2, IFITM1, RAB13, GGPS1, HMGCL, S100A4, PHYH, SAT1, BLVRA, RAB32, C9orf16, DHRS12, MYL5, SPA17, OCEL1, FKBP1B, ABI2, CD46, TNFRSF1A, VAT1, DUSP6, IFI35, TST, ECI1, TP53I3, NEK3, SERPINB6, USP32, IFITM3, MYO1B, SUCLG2, RRAS, YIPF3, ITM2B, ARMCX3, SIL1, MSRB2, PLEKHA1, FBXO2, DECR2, EFHC1, SLC25A21, CRYL1, CORO1B.

## RESULTS

### scRNA-seq of cancer cells at different time points after drug treatments

**Figure 1.**
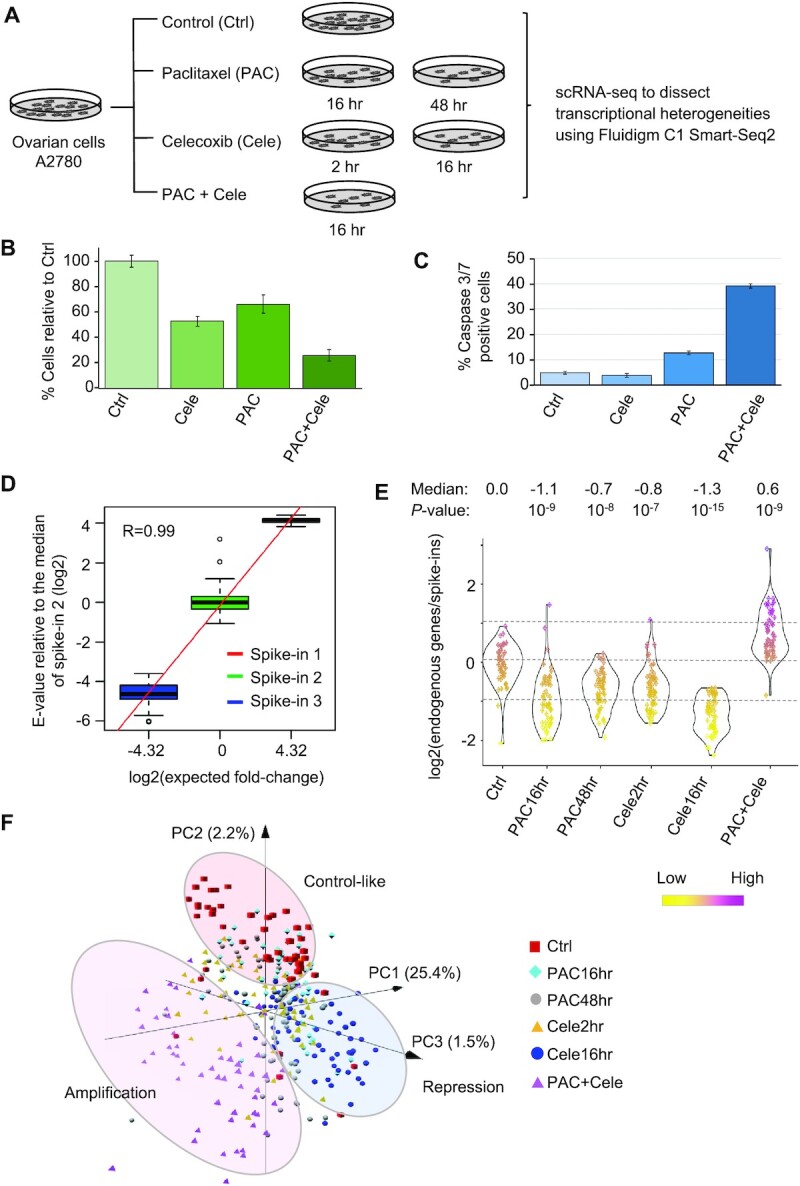
scRNA-seq to examine transcriptional heterogeneities after drug treatment. (**A**) We treated A2780 ovarian cancer cells with paclitaxel (PAC), celecoxib (Cele) and the combination of the two drugs, and collected the cells at different time points for scRNA-seq using Smart-Seq2 on the Fluidigm C1 system. (**B**) The relative cell growth after 48 h drug treatments as compared to controls. (**C**) The fraction of apoptotic cells (caspase 3/7+) after 48 h drug treatment. (**D**) The correlation between expected fold differences of spike-in molecules versus those calculated by scRNA-seq data across single cells. The expression values were normalized to the median of spike-in 2. The Pearson correlation coefficient is indicated in the plot. (**E**) The relative transcriptomic abundance across single cells is measured as the ratio between the number of reads mapped to endogenous mRNAs and the number mapped to the spike-in molecules. The log2(ratio) values were normalized to the median of the control cells. Each dot in the plot represents a single cell. The Wilcoxon rank sum test *P*-values comparing drug treatment groups versus the control are shown. (**F**) Principal component analysis of single cells treated with drugs at different time points. We used expression profiles of genes showing differential expression in at least one drug treatment condition for this analysis. The percentage of variance explained by the principal component is shown in parentheses.

We treated A2780 ovarian cancer cells with paclitaxel, celecoxib and their combination (Figure 1A). Consistent with previous reports, co-treatment of celecoxib increased paclitaxel-induced apoptosis (Figure 1B and C). To characterize intercellular transcriptional heterogeneity mediating the drug response, we performed full-length scRNA-seq with Smart-seq2 using the Fluidigm C1 system for a total of 372 live cells at different time points after drug treatment, including control (57 cells), 16 h (58 cells) and 48 h (65 cells) with paclitaxel, 2 h (66 cells) and 16 h (67 cells) with celecoxib, and 16 h with the two drugs (59 cells) (Figure 1A and Supplementary Figure S1A). We used Smart-seq2 for the experiment because it has the highest sensitivity to capture expressed transcripts compared to other scRNA-seq techniques (31). We picked the early time points because initial gene expression changes after drug treatment are crucial for determining cell fates (32), and they are representative of the dynamic chromatin packing density revealed by our PWS experiments. To examine the regulation of global transcriptomic abundance in single cells and perform quality control of the scRNA-seq experiment, we used a pre-designed spike-in set consisting of three polyA-tailed RNAs with variable lengths and concentrations (Supplementary Table S1). We added an equal volume of the spike-in set to each well of the 384-well plate during the library preparation using the Fluidigm C1 system.

For each single-cell library, sequencing reads were mapped to the hybrid reference transcriptome (GENCODE-defined endogenous transcripts + spike-in RNA sequences) to quantify the RNA expression. The fold expression differences of spike-in RNAs are consistent with the pre-designed conditions (Figure 1D and Supplementary Figure S2), indicating that our scRNA-seq experiment quantitatively measures RNA expression levels. For each endogenous gene, we normalized its ‘transcript per million’ (TPM) value to that of the highest expressed spike-in RNA in the same cell and used the normalized value to indicate its expression level. We removed 34 poor quality cells with a low number of housekeeping genes detected (Supplementary Table S2, Supplementary Figure S1B and see Materials and Methods section for details) (21,22). We retained 338 single cells for further analyses. In addition, we required that a gene included in the analyses should be detected in at least 30% of single cells from at least one experimental condition. After these quality-control steps, we were able to study the dynamic expression of ∼9600 genes.

### Dynamic regulation of global transcriptomic levels

To measure the relative transcriptomic amount in a single cell, we calculated the ratio between the number of reads mapped to endogenous mRNAs and the number mapped to spike-in RNAs. In each experimental condition, the overall mRNA abundance varied >3-fold across single cells indicating intercellular heterogeneity (Figure 1E) (33). If we consider the median value across single cells in a condition, the global transcriptomic level is repressed after paclitaxel or celecoxib treatment alone. As compared to the control cells, the overall mRNA amount decreases ∼2.1-fold at 16 h of paclitaxel treatment (*P* < 10^−9^), and reduces ∼2.4-fold at 16 h with celecoxib (*P* < 10^−15^) (Figure 1E). Interestingly, after 16 h co-treatment of paclitaxel and celecoxib, the transcriptomic amount was up-regulated ∼1.5-fold (*P* < 10^−9^) (Figure 1E). These results suggest that the combination of the two drugs switches the transcriptional repression induced by a single drug to the transcriptional amplification state. The same result was produced when we used a different computational approach to measure relative transcriptomic levels, by calculating the ratio between the sum of TPM values of the top 5000 expressed mRNAs vs. that of the highest spike-in RNA (Supplementary Figure S3).

Considering averaged expression values across single cells, spike-in normalization revealed that 2571 genes were down-regulated >1.5-fold after 48 h of paclitaxel treatment and 4241 genes were down-regulated with 16 h of celecoxib, while very few (<90 genes) showed upregulation (Supplementary Figure S4A). With the combination of two drugs, 2936 genes were up-regulated >1.5-fold and only 170 genes were down-regulated (Supplementary Figure S4A). If examining differential gene expression using TPM values without spike-in normalization, we obtained more balanced numbers of up-regulated and down-regulated genes (Supplementary Figure S4BC). The results confirmed that spike-in normalization is essential for identifying the regulation of global transcriptomic abundance.

Besides drug-induced expression changes, we examined how intercellular variances of single cells within a treatment group contribute to expression differences among the cells. For each treatment condition, we identified genes whose expression variance (measured as coefficient of variation) greater than those expressed at similar levels (see Materials and Methods for detail). Only 68–100 genes within each group showed high expression variance (Supplementary Figure S5A and Supplementary Table S3). These genes tend to reoccur among treatment groups (Supplementary Figure S5BC). Gene ontology analyses showed that they are enriched in the mitotic cell cycle pathway (*P*-value < 10^−15^). This presumably reflects that single cells are in different cell cycle phases (34,35). Compared to the drug-induced gene expression changes, the intercellular expression heterogeneity within a treatment group is much smaller. We focused on analyzing drug-induced gene regulation in the following analyses.

### A random forest model classifies single cells into transcriptional repression, amplification and control-like states

Next, using the principal component analysis, we clustered single cells based on their transcriptomic profiles. The unbiased clustering showed that the single cells are likely in three different states: the control-like state (e.g. untreated cancer cells), the transcriptional repression state (e.g. cells with 16 h celecoxib treatment) and the transcriptional amplification state (e.g. cells co-treated with paclitaxel and celecoxib) (Figure 1F). Cells from other conditions, especially with paclitaxel treatment alone, consist of mixtures corresponding to different states (Figure 1F).

Additionally, using out-of-bag estimates, we measured the prediction power of random forest to classify single cells from each drug treatment group versus those from other groups (Supplementary Figure S6). We observed low classification error rates for untreated cancer cells, cells with 16 h celecoxib and those co-treated with two drugs, indicating that cells from these treatment groups tend to have unique gene signatures and exist in homogenous states. Paclitaxel-treated cells have higher out-of-bag error rates, indicating that they are likely to compose with cells from mixed states. These results are consistent with the findings from the above principal component analysis.

**Figure 2.**
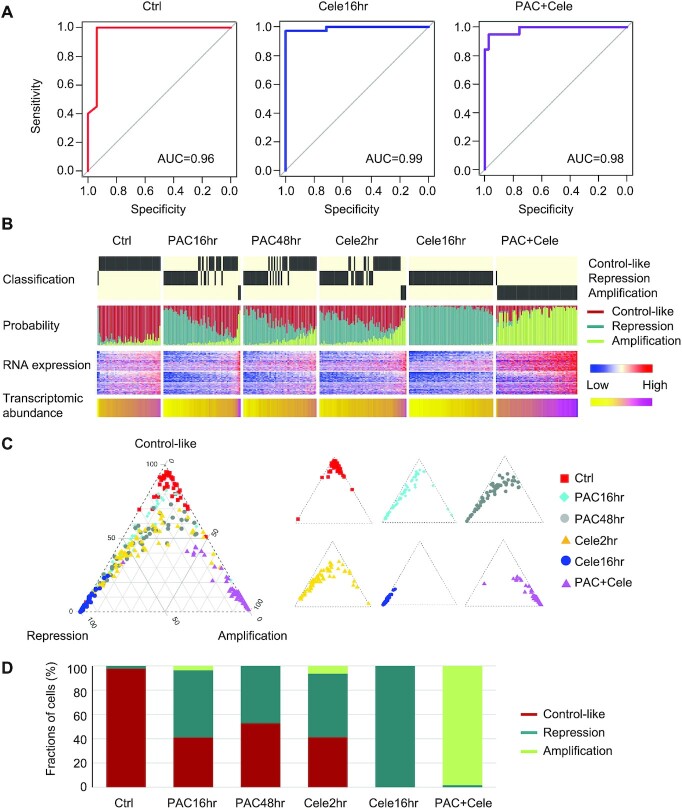
A random forest model classifies single cells into three different states: transcriptional repression, amplification and control-like. (**A**) We used transcriptomic profiles of cells with 16 h of celecoxib, 16 h of combination treatment and control cells to represent the transcriptional repression, amplification and control-like states, respectively. We used 2/3 of single cells for training and the remaining 1/3 of cells for testing. The algorithm performance when classifying the three states based on the testing set was measured using ROC curves, and the area under the ROC curves (AUC) is shown in each plot. (**B**) Classifying single cells into three states based on their gene expression profiles using the random forest model. For each single cell, the transcriptomic abundance (as in Figure 1E), RNA expression profile (normalized *E*-values by mean of control cells), predicted *P*-values of the three states, and the decision state are shown. Each column in the plot represents a single cell. (**C**) The triangle plot showing the predicted *P*-values of the three states across single cells. The pooled single cells are shown on the left, and cells with different treatment conditions are shown on the right. (**D**) Fraction of cells classified into the three states under different drug treatment conditions.

To further characterize the intercellular heterogeneity among the 338 single cells, we developed a supervised learning model using the random forest method to classify single cells into three states based on their transcriptomic profiles. We used gene expression profiles of cells treated with celecoxib for 16 h, cells co-treated with the two drugs and untreated cancer cells to represent transcriptional repression, amplification and control-like states, respectively, because the cells from these conditions tend to be homogenous (Figure 1F and Supplementary Figure S6). We used expression profiles from a randomly selected 2/3 of cells in each group as the training set, and profiles of the remaining 1/3 as the testing set to evaluate the algorithm performance. The random forest model performed the feature gene selection and classified cell states with high accuracy (area under the receiver operating characteristic [ROC] curve [AUC] >0.95 for each of three states) (Figure 2A). We then applied the random forest model and classified all single cells based on their RNA expression profiles (Figure 2B and C).

And our classification results were robust if we used an alternative approach to pick training sets by randomly selecting single cells located in the three angles based on our principal component analysis (Figure 1F) without restricting the preexisting sample labels (see Materials and Methods section for detail). The new method resulted in ∼93.8% consistent classified cell states compared to the method described above (the 90% confidence interval is [90.5%, 95.9%]),

Our random forest classification process refined the dynamic compositions of cells from different states after paclitaxel treatment. At 16 h with paclitaxel, 55% of cells show transcriptional repression, 4% show transcriptional amplification and 41% are control-like (Figure 2D). At 48 h, cells treated with paclitaxel remain in a mixture of states, including 47% in the transcriptional repression state and 53% in the control-like state. These results highlight the intercellular transcriptional heterogeneity in response to paclitaxel and are consistent with our PWS microscopy results showing changes in chromatin packing density (14).

### Regulation of OXPHOS and cell cycle genes is a major determinant of transcriptomic state

**Figure 3.**
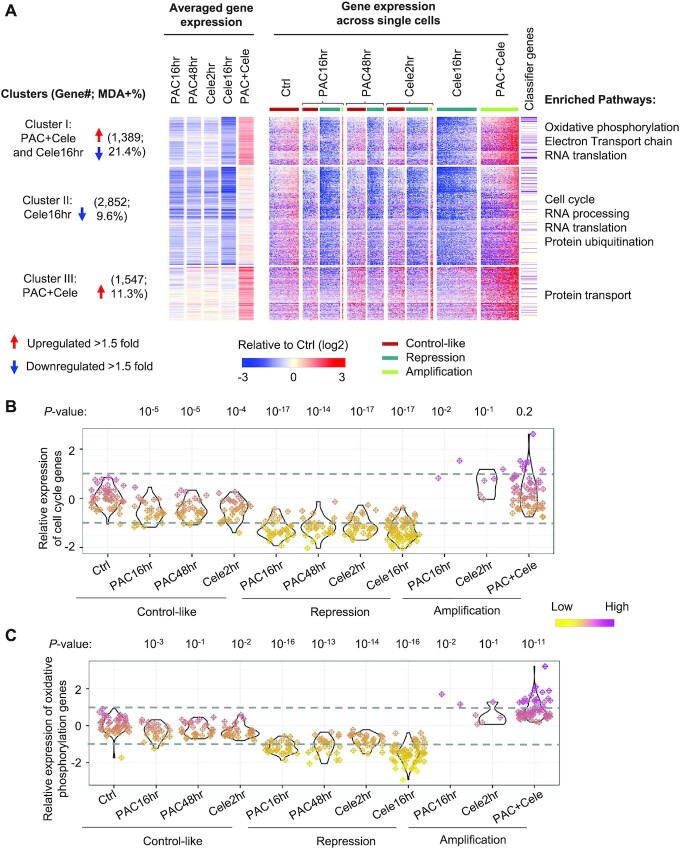
OXPHOS and cell cycle genes are major classifiers to define the cell states. (**A**) The heatmap showing the differentially expressed gene clusters based on their relative changes after drug treatment. The gene expression values were normalized to the mean of the control cells. Single cells in each treatment condition were further grouped by their defined states. The classifier genes in the random forest model with MDA value >0 are indicated in the heatmap. The enriched pathways in each cluster based on gene ontology analyses are shown. (**B**) The relative expression of cell cycle pathway genes across single cells, which were grouped based on drug treatment conditions and defined states. Each dot in the plot represents a single cell. The Wilcoxon rank sum test *P*-values comparing drug treatment groups versus the control are shown. (**C**) Similar to (B), the relative expression of OXPHOS pathway genes across single cells, which were grouped based on drug treatment conditions and defined states.

In addition to the global regulation of transcriptomic abundance, individual genes show variable expression changes after drug treatment. We partitioned drug-responsive genes into three co-regulatory clusters based on their relative expression across single cells (Figure 3A and Supplementary Table S4). For each gene, we annotated its contribution to the random forest model indicated by the mean decrease in accuracy (MDA) value (Figure 3A and Supplementary Table S5). A total of 2852 genes (cluster II in Figure 3A) were down-regulated (>1.5-fold) in cells showing transcriptional repression and were not regulated in other conditions. These genes are enriched in gene ontology pathways such as ‘cell cycle’, ‘RNA processing’ and ‘protein ubiquitination’ (*P* < 10^−24^; Supplementary Figure S7; Figure 3A and B). The down-regulation of cell cycle genes indicates that the single-agent treatment inhibited cell proliferation. Especially, celecoxib treatment alone did not induce cell apoptosis, and the inhibition of cancer cell growth resulted from homogeneous inhibition of cell cycle genes across single cells (Figure 1B and C). Next, we examined whether the drug treatment modulated the expression of genes regulating a particular cell cycle phase. To this end, we analyzed signature genes uniquely expressed in the G1/S, S, G2 and G2/M phases, respectively (27). These genesets showed a significant positive correlation of differential expression across single cells upon drug treatment (Supplementary Figure S8). Although paclitaxel-treated cells are arrested in the G2/M phase (7), the inhibition of cell cycle genes is not limited to the particular phase.

A total of 1389 genes were down-regulated (>1.5-fold) in cells showing transcriptional repression by single drugs but were up-regulated (>1.5-fold) upon combination treatment (cluster I in Figure 3A). These genes are enriched with MDA values >0 in the random forest model (Figure 3A) and contribute most significantly to cell state classification. Gene ontology analyses showed that they are enriched in pathways such as ‘OXPHOS’, ‘generation of precursor metabolites and energy’, and ‘RNA translation’ (*P* < 10^−22^; Figure 3C and Supplementary Figure S7). Unexpectedly, co-treatment of paclitaxel and celecoxib activated many genes regulating mitochondrial-related functions and the OXPHOS pathway. We further analyzed the genesets encoding complex I, II, III, IV and ATP synthase of the electron transport chain. These genesets were synchronically regulated across single cells, suggesting a coherent transcriptional module regulates their expression during drug response (Supplementary Figure S9). These results suggest that co-treatment with paclitaxel and celecoxib triggers a transcriptional amplification program activating OXPHOS, which can induce the generation of reactive oxygen species in cancer cells and promote cell apoptosis (36).

### Metabolism and inflammation genes are activated in a subpopulation of paclitaxel-treated cancer cells

**Figure 4.**
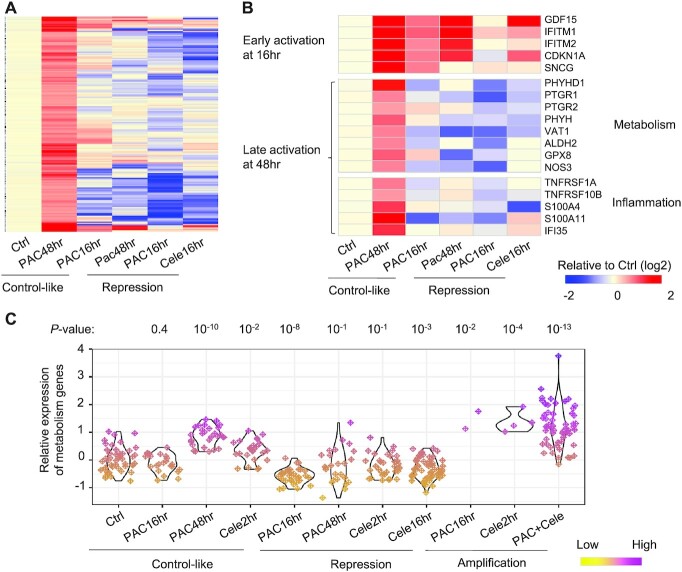
Genes activated in the control-like subpopulation of cells after 48 h of paclitaxel treatment. (**A**) Heatmap showing the expression changes of 177 genes activated in control-like cells after 48 h of paclitaxel treatment. Single cells were grouped based on drug treatment conditions and defined states. For each group, the averaged expression values of a gene across single cells were used to indicate its expression level. The expression fold changes compared to the control cells are shown in the heatmap. (**B**) Example paclitaxel-activated genes showing early activation at 16 h of paclitaxel treatment, and those showing late activation at 48 h and in pathways regulating metabolism and inflammation. (**C**) The relative expression of the metabolism genes across single cells grouped based on treatment conditions and defined states. The paclitaxel-activated genes in the GO:0055114: oxidation-reduction process were used for the analyses. The Wilcoxon rank sum test *P*-values comparing drug treatment groups versus the control are shown.

At 48 h of paclitaxel treatment, 53% of cells showed global transcriptomic abundance comparable to untreated cells (Figure 2D). This subpopulation of cells did not fully mimic untreated cells but showed unique gene signatures with 177 genes up-regulated by >1.5-fold (Figure 4A and Supplementary Table S6). Among these, 20 genes show early activation (>1.5-fold) at 16 h, such as the interferon-induced transmembrane proteins IFITM1 and IFITM2, a driver of p53-dependent cell cycle arrest p21 (CDKN1A), and the growth differentiation factor-15 (GDF-15) (Figure 4B).

The 157 other genes showed late activation at 48 h. The gene ontology analyses showed that they are enriched in genes regulating cellular metabolism (*P* < 10^−9^ for the pathway ‘oxidation-reduction [redox] process’) (Figure 4B and C). These include enzymes regulating lipid metabolism (e.g. ACACA, PTGR1 and SCD) and carboxylic acid biosynthesis (e.g. PHGDH and ASNS). In addition, some pro-inflammatory molecules, such as TNFRSF1A, IFI35, SERPINF1, IFNAR2 and S100A4, were up-regulated (Figure 4B). These results suggest that a subpopulation of cells rewired their intrinsic metabolic and inflammatory pathways after 48 h of paclitaxel treatment.

### A paclitaxel response index predicts treatment efficacy across several hundred cancer cell lines and in breast cancer patients

**Figure 5.**
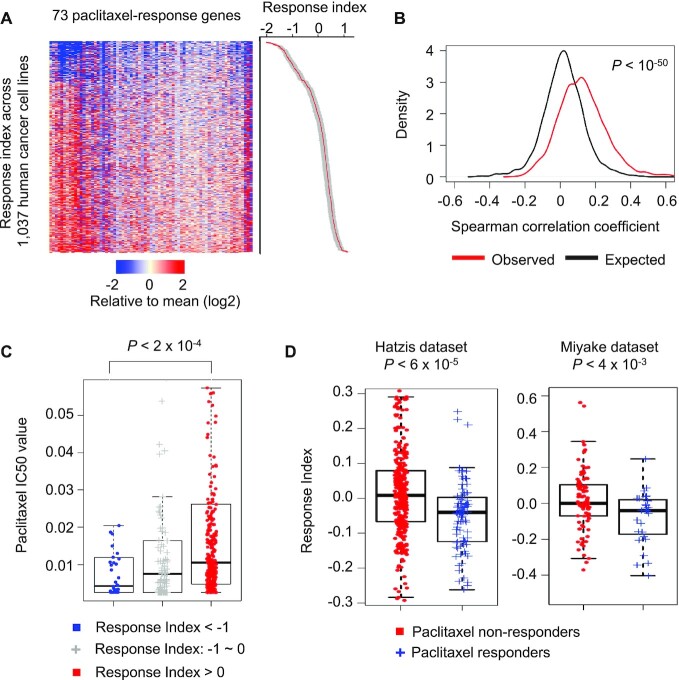
A gene module consisting of paclitaxel-activated genes predicts paclitaxel efficacy across cancer cell lines and breast cancer patients. (**A**) 73 paclitaxel-activated genes showed a positive correlation of expression across 1037 cancer cell lines. We developed a paclitaxel response index to quantitatively measure the relative expression of these genes in a sample (standard error values are shown in gray). (**B**) Distribution of the observed and expected Spearman correlation coefficients of the expression levels between the gene pairs. The observed values were calculated based on gene pairs in (A). The expected correlation values were calculated by randomly picked gene pairs expressed in the cancer cell lines. The Wilcoxon rank sum test *P*-value comparing the observed versus expected correlation coefficients is shown. (**C**) Cancer cell lines were grouped based on the paclitaxel-response index values in (A). Then, we compared their paclitaxel IC_50_ values. The Wilcoxon Rank-Sum test *P*-value comparing two indicated groups is shown in the plot. (**D**) Breast cancer patients from two cohorts were grouped based on their clinical response to paclitaxel treatment. Then, we compared the paclitaxel response index between the two groups of patients. The Wilcoxon Rank-Sum test *P*-values comparing two groups are shown in the plot.

Next, we examined whether the activated inflammatory and metabolic gene signature plays a regulatory role in paclitaxel efficacy. We reasoned that if these genes function as a regulatory module, their co-activation should not be unique to paclitaxel response but should be general across biological conditions because genes regulating a biological process tend to form a coexpression network (37,38). To this end, we examined the co-expression of the 177 paclitaxel-activated genes across 1037 cancer cell lines from 26 primary tissue types using data from the Cancer Cell Line Encyclopedia (CCLE) database (28). By developing an iterative computational method, we found that a gene module consisting of 73 genes (41.2% of the total) showed a significant positive correlation of expression with each other (see Materials and Methods section for details) (*P* < 10^−50^ compared to expected distribution; Figure 5A and B). The metabolic and inflammatory genes described above were present in this gene module. The data also indicate that the paclitaxel-induced gene module is intrinsically active in many cancer cells.

As CCLE also measured the half-maximal inhibitory concentration (IC_50_) values of paclitaxel in these cancer cell lines, we examined whether there is a correlation between the gene module expression and the IC_50_ values. Based on the relative expression of the genes in the module, we developed a paclitaxel response index to quantify the geneset expression, which was calculated as the median of the normalized expression values of the 73 genes (Figure 5A). Next, we grouped cell lines based on their paclitaxel response index. Cancer cells with greater index values tended to show higher paclitaxel IC_50_ values (*P* < 2 × 10^−4^, Wilcoxon rank-sum test; AUC of the ROC curve = 0.653; Figure 5C, Supplementary Figure S10A and Supplementary Table S7). These data indicate that higher baseline expression of the gene module is linked with decreased paclitaxel efficacy in cancer cells.

Furthermore, we examined the impact of the gene module on paclitaxel response across breast cancer patients. We analyzed the RNA expression and clinical data from two published breast cancer patient cohorts (29,30). Indeed, responders to paclitaxel treatment showed lower paclitaxel response index values compared to non-responders (*P* < 4 × 10^−3^, Wilcoxon rank-sum test; AUC of the ROC curve = 0.66; Figure 5D and Supplementary Figure S10B–E, Supplementary Table S8). The AUC values are comparable to those obtained previously using other computationally identified gene module markers (39). These results further indicate that our paclitaxel response index can predict the clinical outcome of paclitaxel treatment.

## DISCUSSION

Using the Fluidigm C1 Single-Cell Auto Prep System, we added a constant amount of the spike-in RNA set to each well during library preparation. During the data analyses, we calculated gene expression levels by aligning sequencing reads to a hybrid transcriptome combining genome-encoded transcripts and spike-in RNAs. The fold differences of spike-in RNAs learned from the sequencing data are in accordance with the pre-designed concentrations, indicating that our scRNA-seq quantitatively measures RNA levels. Furthermore, by normalizing endogenous gene expression to that of spike-in RNAs, we found that mRNA abundance is drastically differentially regulated across single cells after drug treatment. These differences would not be observed in measurements using TPM or RPKM (reads per kilobase of transcript per million reads mapped) values for endogenous genes alone, as these calculations rely on the presumption that global transcriptomic levels are comparable across cells. Our results confirm the value of adding the synthetic spike-in RNAs for scRNA-seq.

One of the major findings of this study is that drug treatments induce variable changes in global mRNA abundance in a cell. This level of regulation has been commonly overlooked by previous studies due to the lack of gene expression normalization using spike-in RNAs for RNA-seq experiments. Interestingly, paclitaxel treatment alone induces transcriptional repression in a subpopulation of cells. The down-regulation of cell cycle genes is consistent with the mitotic arrest induced by paclitaxel. Here, we found that genes expressed in the G1/S, S, G2 and G2/M phases are synchronically down-regulated at comparable levels, indicating the inhibition of cell cycle genes is not specific to a particular phase. A coordinated transcriptional network is likely to regulate the expression of cell cycle genes.

**Figure 6.**
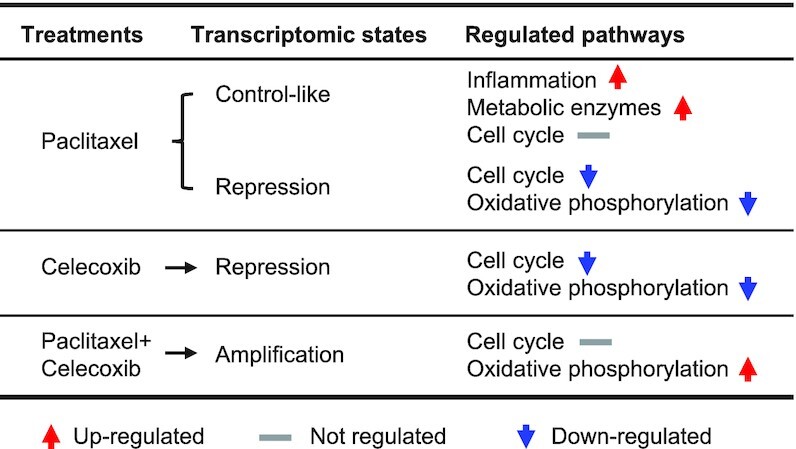
The table shows the transcriptomic states and regulation of associated pathways after the various drug treatments.

Interestingly, the combination of paclitaxel and celecoxib induces transcriptional amplification, which is opposite to the transcriptional repression caused by the single drug treatments (Figure 6). The fact that the expression of cell cycle genes is unchanged after co-treatment with the two drugs suggests that another regulatory pathway promotes cell apoptosis in this context. Unexpectedly, the OXPHOS pathway showed the most significant up-regulation. OXPHOS is the major process to generate mitochondrial reactive oxygen species (ROS). Hyperactivation of OXPHOS can cause leakage of electrons from electron transport chains, leading to a partial reduction of oxygen and the formation of superoxide in cells (40,41). The resulting ROS production could be the mechanism by which the drug combination enhances apoptosis and cell-killing efficiency. These results also indicate that the interplay between two drugs can trigger a novel transcriptional program, increasing the efficacy.

Dynamic global transcriptional activity can result from the regulation of the cellular amount of RNA polymerase II (Pol II), the efficiency of Pol II recruitment to promoters or the rate of transcriptional elongation (20,42). These molecular mechanisms have been characterized in a few biological processes. Cancer cells with overexpressed MYC show higher transcriptomic abundance than normal cells because MYC located in promoters and enhancers can directly recruit the transcriptional elongation factor P-TEFb, resulting in transcriptional amplification (43). In B-cell acute lymphoblastic leukemia, overexpression of the nucleosome remodeling protein HMGN1 suppresses heterochromatin marker H3K27me3 levels and promotes B cell transcriptomic levels and proliferation (44). Future efforts are needed to identify the molecular regulators mediating transcriptional amplification or suppression after drug treatment.

The initial expression changes of cancer cells in response to drugs are important in determining their fate. The probability of drug-induced apoptosis appears to be stochastic from the perspective of a single cell but has a fixed ratio in the larger cell population (45–47). The pre-existing gene expression programs regulate the ratio of killed cells (i.e. the IC_50_ value of a drug). At the single-cell level, cell death is determined by whether pro-apoptotic signals have accumulated to the threshold level. Here, we found that transcriptomic abundance shows intercellular heterogeneity in response to paclitaxel treatment. This heterogeneity is a new regulatory layer contributing to chemoresistance. In about half of the treated cells, the global transcriptional levels are comparable to the control state. Unexpectedly, a coherent gene module consisting of a small number of genes (73 genes) was activated in a subpopulation of paclitaxel-treated cells, and these genes were highly enriched in redox and inflammatory pathways. We showed that the baseline expression of this gene module predicts chemo-response *in vitro* and *in vivo*. The metabolic switch in cancer cells is known to play a major regulatory role during drug response (48,49). For example, some genes in the module, such as aldehyde dehydrogenase (ALDH2) and acetyl-CoA carboxylase (ACACA), are known oncogenes, promoting cancer stem cell formation and drug resistance (50,51). Future experiments tracing the metabolic changes of cancer cells after drug treatment will provide further functional insights into this level of regulation. Notably, our analyses were performed using the cultured cancer cells and the gene signatures we obtained did not account for molecular interactions between cancer cells and the immune microenvironment. Further work using single-cell experiments tracing paclitaxel efficacy in immune-competent models can reveal additional regulatory layers of the resistance mechanisms.

## DATA AVAILABILITY

The sequencing datasets generated during the current study are available in the Gene Expression Omnibus (GEO) repository with the accession number GSE162256. Computational codes are available upon request.

## Supplementary Material

lqab054_Supplemental_Files
