# Correction to ‘Spike-in normalization for single-cell RNA-seq reveals dynamic global transcriptional activity mediating anticancer drug response’

**DOI:** 10.1093/nargab/lqad046

**Published:** 2023-05-17

*NAR Genomics and Bioinformatics*, Volume 3, Issue 2, June 2021, lqab054, https://doi.org/10.1093/nargab/lqab054

In the originally published version of this manuscript, the following grant was inadvertently omitted from the Funding section:

‘NIH Grant R01CA228272’.

These details have been corrected only in this correction notice to preserve the published version of record.

